# A pan-cancer analysis of the role of USP5 in human cancers

**DOI:** 10.1038/s41598-023-35793-2

**Published:** 2023-06-02

**Authors:** Bokang Yan, Jiaxing Guo, Shuang Deng, Dongliang Chen, Meiyuan Huang

**Affiliations:** 1 Department of Pathology, Zhuzhou Hospital Affiliated to Xiangya School of Medicine, Central South University, Zhuzhou, 412007 Hunan China; 2 Department of Hematology, Zhuzhou Hospital Affiliated to Xiangya School of Medicine, Central South University, Zhuzhou, 412007 Hunan China

**Keywords:** Tumour biomarkers, Cancer genetics, Epigenetics

## Abstract

Posttranslational modifications (PTM) such as acetylation, deubiquitination, and phosphorylation of proteins, play important roles in various kinds of cancer progression. Ubiquitin-specific proteinase 5 (USP5), a unique member of deubiquitinating enzymes (DUBs) which recognizes unanchored polyubiquitin specifically, could regulate the stability of many tumorigenesis-associated proteins to influence cancer initiation and progression. However, the diverse biological significance of USP5 in pan-cancer has not been systematically and comprehensively studied. Here, we explored the role of USP5 in pan-cancer using The Cancer Genome Atlas (TCGA) and Genotype-Tissue Expression (GTEx) database, and we also acquired and analyzed data via various software and web platforms such as R, GEPIA2.0, HPA, TISIDB, cBioPortal, UALCAN, TIMER 2.0, CancerSEA and BioGRID. USP5 expression was high in most cancers and differed significantly in different molecular and immune subtypes of cancers. In addition, USP5 had certain diagnostic value in multiple cancers, and high expression of USP5 generally predicted poor prognosis for cancer patients. We also found that the most frequent genetic alterations type of USP5 was mutation, and the DNA methylation level of USP5 decreased in various cancers. Furthermore, USP5 expression correlated with cancer-associated fibroblasts (CAFs), endothelial cells (EC) and genetic markers of immunodulators in cancers. Moreover, the result from single cell sequencing showed that USP5 could regulate several tumor biological behaviors such as apoptosis, DNA damage and metastasis. Gene enrichment analysis indicated “spliceosome” and “RNA splicing” may be the critical mechanism for USP5 to involve in cancer. Taken together, our study elucidates the biological significance of USP5 in the diagnosis, prognosis and immune in human pan-cancer.

## Introduction

Cancer is a leading cause of human death, with significant negative impacts on social health and the economy all over the world^1,2^. Although the development of surgery, chemoradiotherapy, targeted therapy, and immunotherapy have improved the therapeutic effect on cancer, the prognosis and survival rate of cancer patients still remains poor for many reasons, including drug resistance and side effects^3^. Therefore, the identification of new pan-cancer biomarkers and therapy targets for cancer is critical for improving human health ^4^.

Deubiquitination is a common posttranslational modification (PTM) of proteins, which is involved in regulating various physiological functions and pathological processes, such as signal transduction and cancer progression^5,6^. Ubiquitin-specific proteinase 5 (USP5) which is discovered and purified by Wilkinson et al., also known as Ubiquitin isopeptidase T (ISOT), belongs to the ubiquitin-specific proteinase (USP) family, the largest family of deubiquitinating enzymes (DUBs)^7^. USP5 is located near the CD4 gene on human chromosome 12p13 and encodes five separate domains with the size of 93.3-kDa^8^. It is unique in that it can specifically identify and remove ubiquitin from the proximal end of the unanchored polyubiquitin chains^9^. And many researches prove that USP5 regulates a variety of cellular activities, including the repair of DNA double-strand breaks^10^, inflammatory responses^11^, and stress responses^12^.

As for the role of USP5 in cancer, it has attracted the attention from many researchers recently. The functional link between the aberrant USP5 expression and the development of various cancers such as breast cancer^13^, lung cancer^14,15^, colorectal cancer^16^ and hepatocellular carcinoma^17^ has been established by many studies. Meanwhile, USP5 has been demonstrated to be closely correlated with some key molecules and pathways regulating cancer, which indicates the potential value of USP5 as a novel treatment target for cancer^18^. However, the detailed role of USP5 in pan-cancer remains elusive so far.

Nowadays, the rapid development of biological databases makes the bioinformatics analyses much more reliable and representative with large sample size and advanced algorithms. In this study, we explored USP5 expression profiles, diagnostic value, prognostic value, genetic alteration, protein methylation level, immune infiltration, functional states and functional enrichment in pan-cancer by using multiple bioinformatics methods. And this thorough analysis revealed the certain value of USP5 in the diagnosis and prognosis of various cancers, the potential role of USP5 in some unexplored cancers, the underlying molecular mechanisms of USP5 in the pathogenesis of human cancers and the implications of USP5 in anti-tumor immune response.

## Materials and methods

### Gene expression analysis

Human Protein Atlas (HPA) was used to get the USP5 mRNA and protein expression levels in human organs/tissues. The USP5 mRNA data across 33 cancer types and corresponding paracancer and normal samples were obtained from The Cancer Genome Atlas (TCGA) and Genotype-Tissue Expression (GTEx) databases. R software (v 3.6.3) was used to perform statistical analysis, and the “ggplot2” (v3.3.3) package was used for visualization. The Wilcoxon rank-sum test was used to detect the difference between groups, and p < 0.05 was considered statistically significant. Expression Profiling Interactive Analysis 2 (GEPIA2.0) was used to analyze the correlation between the expression of USP5 and patients’ pathological stage in all TCGA cancers. HPA was further used to evaluate expression differences of USP5 at the protein level.

### USP5 expression in molecular and immune subtypes of cancers

TISIDB database (http://cis.hku.hk/TISIDB/) which composed of many data types to evaluate the interaction between cancer and immune system was used to analyze the relationship between USP5 expression and molecular or immune subtypes in pan-cancer.

### Diagnostic value analysis

The receiver operating characteristic (ROC) curve was used to estimate the diagnostic value of USP5 in pan-cancer, via using the data of the mRNA expression of USP5 in cancer and normal tissues in TCGA and GTEx. Package “pROC” (v1.17.0.1) was used to calculate the ROC curves, and the “ggplot2” (v3.3.3) package was used for plotting. The closer the area under the curve (AUC) is to 1, the better the diagnostic accuracy is. AUC in 0.5–0.7 means low accuracy, AUC in 0.7–0.9 means good accuracy, and AUC in 0.9–1 means high accuracy.

### Survival prognosis analysis

Kaplan–Meier plots conducting by the “survival” (v3.2-10) package was used to explore the relationship between USP5 expression and prognosis [overall survival (OS), disease-specific survival (DSS) and progress-free interval (PFI)] of cancers. The “survminer” (v0.4.9) package was used for visualization. And the forest plot was conducted for summarizing and presenting the results of univariate Cox regression.

### Genetic alteration and DNA methylation analysis

The cBioPortal (https://www.cbioportal.org/) was searched for the gene alternations of USP5 in TCGA PanCancer Atlas Studies. The genetic alterations and mutation site information were explored with the “Oncoprint”, “Cancer Type Summary” and “Mutations” modules. And the effect of the gene alterations of USP5 on clinical prognosis, including progress-free survival (PFS), DSS, disease-free survival (DFS), and OS, for all TCGA cancer types was analyzed in the “Comparison” module. Methylation level of USP5 in cancers and corresponding normal tissues was investigated in the UALCAN database (http://ualcan.path.uab.edu/analysis.html).

### Immunogenomic analyses

Various algorithms, such as EPIC, MCPCOUNTER, QUANTISEQ, TIDE, TIMER and XCELL were applied to analyze the relationship between USP5 expression and immune infiltration levels across all TCGA cancers, using TIMER2.0 tool. And we also investigated the correlations between USP5 expression and immunodulators, MHC molecules, chemokines, and chemokine receptors in pan-cancer from the TISIDB database.

### Single cell sequencing

Using CancerSEA, we explored the correlation between USP5 expression and different functional status of cancer cells at the single cell levels. The threshold for correlation between USP5 and cancer functional status was set at correlation ≥ 0.3 and p value < 0.05. Expression profiles of USP5 at single cells was showed by the T-SNE diagrams.

### Gene enrichment analysis

BioGRID was used to explore potential protein interactions with USP5. GEPIA2.0 was applied to collect the top 100 USP5-correlated genes from all TCGA cancer and normal tissues. Then pairwise gene–gene Pearson correlation analysis was performed between USP5 and the selected genes. Heatmap was used to represent the expression status of selected genes containing the partial correlation (cor) and p value. Gene ontology (GO) and Kyoto encyclopedia of genes and genome (KEGG) enrichment analyses for USP5-correlated genes were conducted via the “clusterProfiler” (v3.14.3) and “org.Hs.eg.db” (v3.10.0) package^19^. “ggplot2” (3.3.3) was used to present the results. p value < 0.05 was considered to be statistically significant.

## Results

### USP5 expression in human organs/tissues and pan-cancer

**Figure 1:**
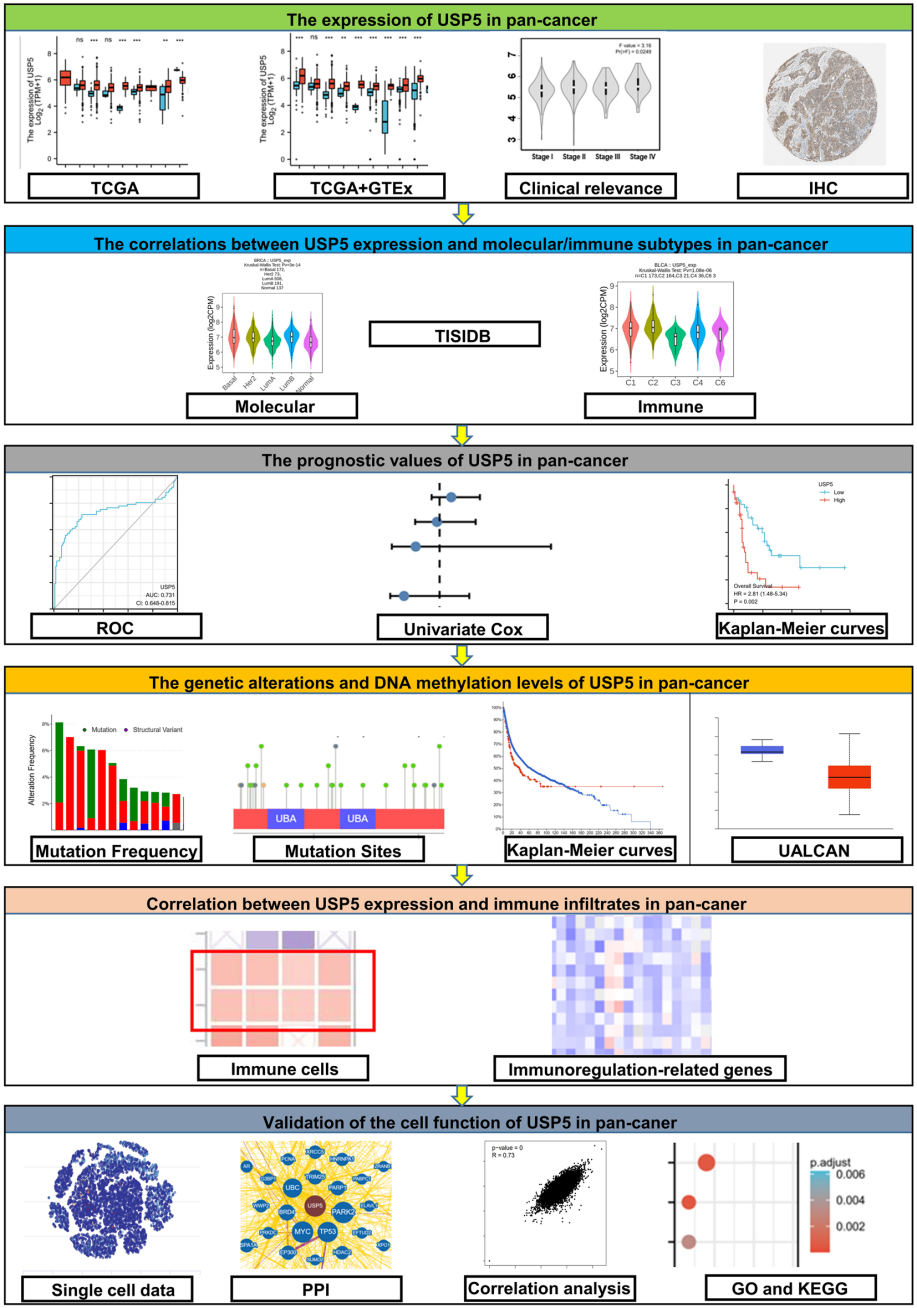
The workflow of this study.

The flowchart of our study was showed in Fig. 1. Initially, we examined the mRNA and protein expression levels of USP5 in various organs or tissues. The results obtained from consensus dataset created by combing the HPA and GTEx transcriptomics datasets showed that mRNA of USP5 mainly expressed in skeletal muscle, skin, tongue, parathyroid gland, testis, pons, esophagus, cerebral cortex, adrenal gland and thymus (Supplementary Fig. 1A,B). In addition, USP5 is primarily expressed in cerebellum, testis, cerebral cortex, hippocampus, caudate, thyroid gland, parathyroid gland, adrenal gland, bronchus and lung demonstrated by the HPA dataset (Supplementary Fig. 1A,C).

**Figure 2:**
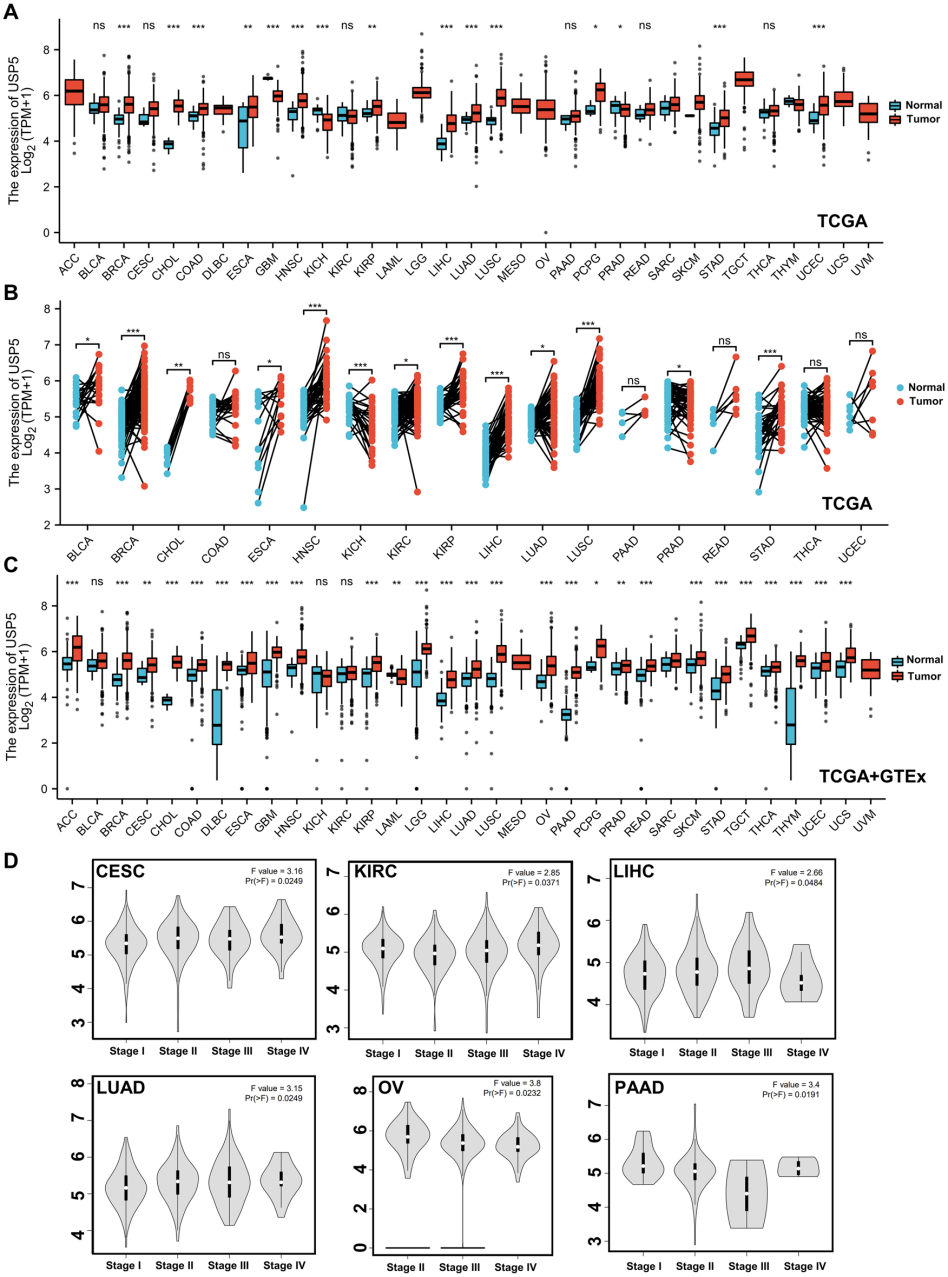
The mRNA expression level of USP5 in pan-cancer. (**A**) Unpaired analysis of USP5 mRNA expression in paracancerous tissues and cancers from TCGA database. (**B**) Paired analysis of USP5 mRNA expression in paracancerous tissues and cancers from TCGA database. (**C**) USP5 mRNA expression level in normal tissues and cancers from TCGA and GTEx databases. (**D**) Analyse of USP5 mRNA expression level by the main pathological stages using GEPIA2.0. *p < 0.05, **p < 0.01, ***p < 0.001. *ns* not significant.

Then, we analyzed the mRNA expression level of USP5 in pan-cancer, and the list of abbreviations for all cancers analyzed were included in Supplementary Table 1. Unpaired analysis of USP5 mRNA expression between paracancerous tissues and cancers revealed that USP5 expressed significantly higher in BRCA (Breast invasive carcinoma), CHOL (Cholangiocarcinoma), COAD (Colon adenocarcinoma), ESCA (Esophageal carcinoma), HNSC (Head and Neck squamous cell carcinoma), KIRP (Kidney renal papillary cell carcinoma), LIHC (Liver hepatocellular carcinoma), LUAD (Lung adenocarcinoma), LUSC (Lung squamous cell carcinoma), PCPG (Pheochromocytoma and Paraganglioma), STAD (Stomach adenocarcinoma) and UCEC (Uterine Corpus Endometrial Carcinoma), and significantly lower in GBM (Glioblastoma multiforme), KICH (Kidney Chromophobe) and PRAD (Prostate adenocarcinoma). There was no obvious difference shown in BLCA (Bladder Urothelial Carcinoma), CESC (Cervical squamous cell carcinoma and endocervical adenocarcinoma), KIRC (Kidney renal clear cell carcinoma), PAAD (Pancreatic adenocarcinoma), READ (Rectum adenocarcinoma) and THCA (Thyroid carcinoma). ACC (Adrenocortical carcinoma), DLBC (Lymphoid Neoplasm Diffuse Large B-cell Lymphoma), LAML (Acute Myeloid Leukemia), LGG (Brain Lower Grade Glioma), MESO (Mesothelioma), OV (Ovarian serous cystadenocarcinoma), SARC (Sarcoma), SKCM (Skin Cutaneous Melanoma), THYM (Thymoma), TGCT (Testicular Germ Cell Tumors), UCS (Uterine Carcinosarcoma) and UVM (Uveal Melanoma) were unable to be analyzed due to the lack of sufficient paracancerous samples (Fig. 2A). Moreover, the paired sample analysis showed that compared with paracancerous tissues, USP5 was overexpressed in BLCA, BRCA, CHOL, ESCA, HNSC, KIRC, KIRP, LIHC, LUAD, LUSC and STAD. On the contrary, USP5 was decreased in KICH and PRAD. And no significantly differential expression of USP5 observed in COAD, PAAD, READ, THCA and UCEC (Fig. 2B). Given the lack of paracancerous tissues in some analysis, we further detected the expression differences of USP5 using the combination of TCGA and GTEx. And the results showed that low expression of USP5 was only observed in LAML, and high expression of USP5 was observed in ACC, BRCA, CESC, CHOL, COAD, DLBC, ESCA, GBM, HNSC, KIRP, LGG, LIHC, LUAD, LUSC, OV, PAAD, PCPG, PRAD, READ, SKCM, STAD, TGCT, THCA, THYM, UCEC and UCS. There was no difference shown in BLCA, KICH and KIRC. Analysis of MESO, SARC and UVM was not possible due to lack of sufficient normal samples (Fig. 2C). Furthermore, we used GEPIA2.0 to explore the effect of USP5 mRNA expression on patient’s pathological stages. And we found that the expression of USP5 was significantly correlated with the pathological stages of CESC, KIRC, LIHC, LUAD, OV and PAAD (Fig. 2D).

**Figure 3:**
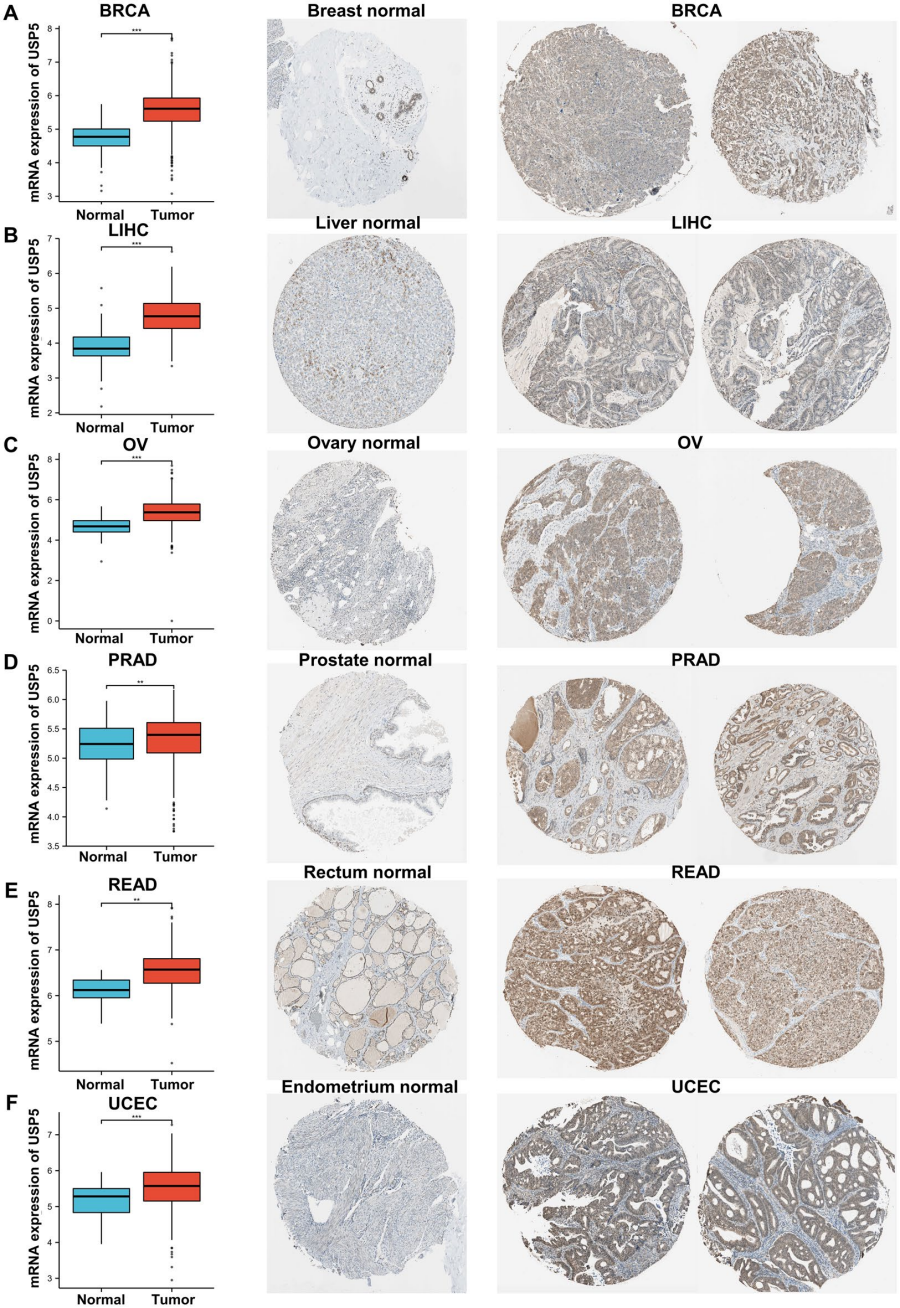
The different expression of USP5 between normal tissues and tumor tissues. (**A**) Breast. (**B**) Liver. (**C**) Ovary. (**D**) Prostate. (**E**) Rectum. (**F**) Endometrium. **p < 0.01, ***p < 0.001. The mRNA expression level of USP5 obtained from TCGA + GTEx and IHC results provided by the HPA dataset.

At last, we further explored the protein expression level of USP5 in pan-cancer using the National Cancer Institute’s CPTAC dataset and the IHC results provided by the HPA dataset. The result of CPTAC analysis indicated that the protein expression of USP5 was up-regulated and correlated with pathological stages in clear cell RCC (renal cell carcinoma) and OV (Supplementary Fig. 2A,B). And the immunochemistry results of the Human protein atlas showed that staining intensity of USP5 was greater in many cancers, mainly including BRCA, LIHC, OV, PRAD, READ and UCEC, which was consistent with the analysis result of the mRNA expression level of USP5 from TCGA + GTEx (Fig. 3A–F). Overall, USP5 was overexpressed in most cancers.

### USP5 expression in different molecular subtypes and immune subtypes of pan-cancer

**Figure 4:**
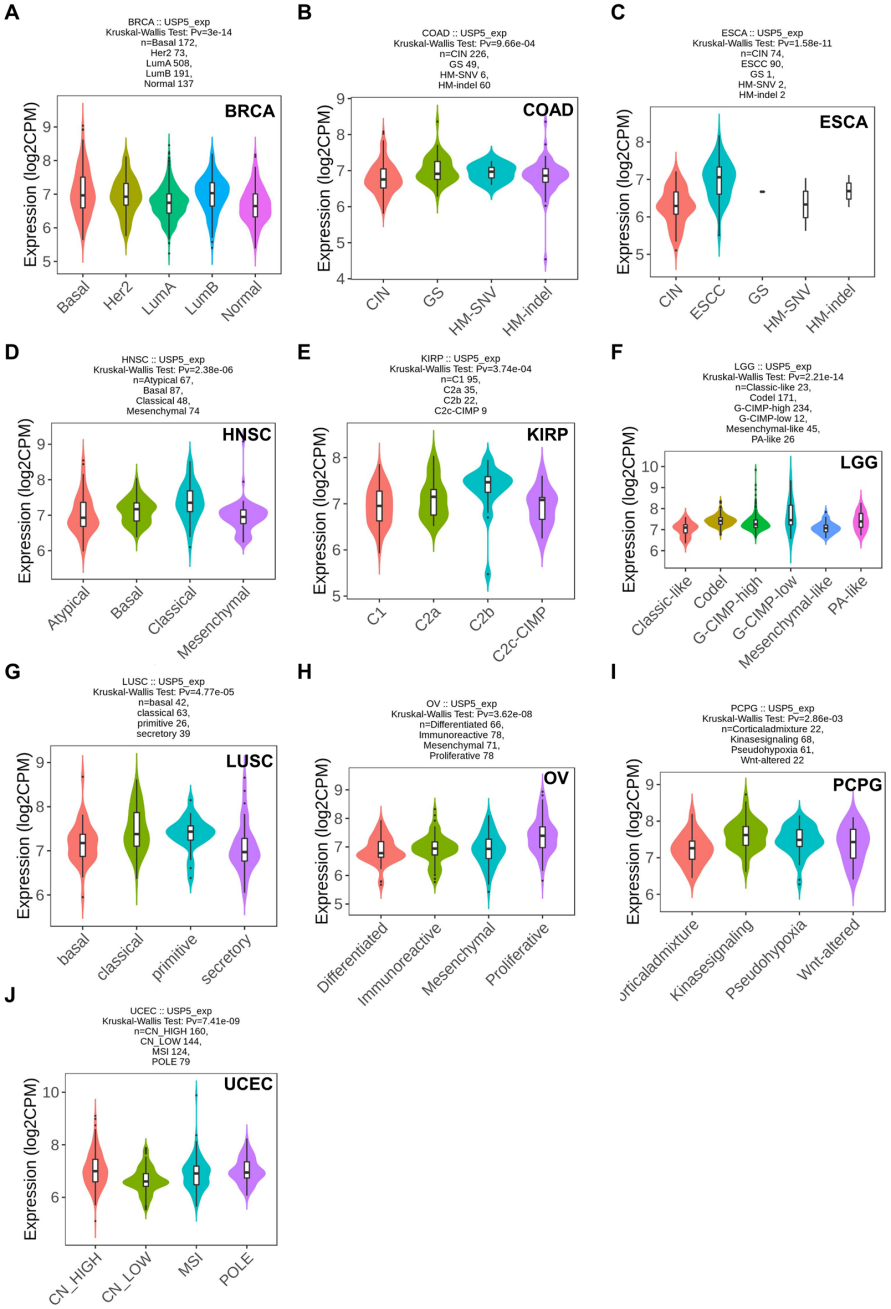
Correlations between USP5 expression and molecular subtypes in pan-cancer. (**A**) BRCA. (**B**) COAD. (**C**) ESCA. (**D**) HNSC. (**E**) KIRP. (**F**) LGG. (**G**) LUSC. (**H**) OV. (**I**) PCPG. (**J**) UCEC. Correlation analysis between USP5 expression and molecular subtypes performed via TISIDB database.

**Figure 5:**
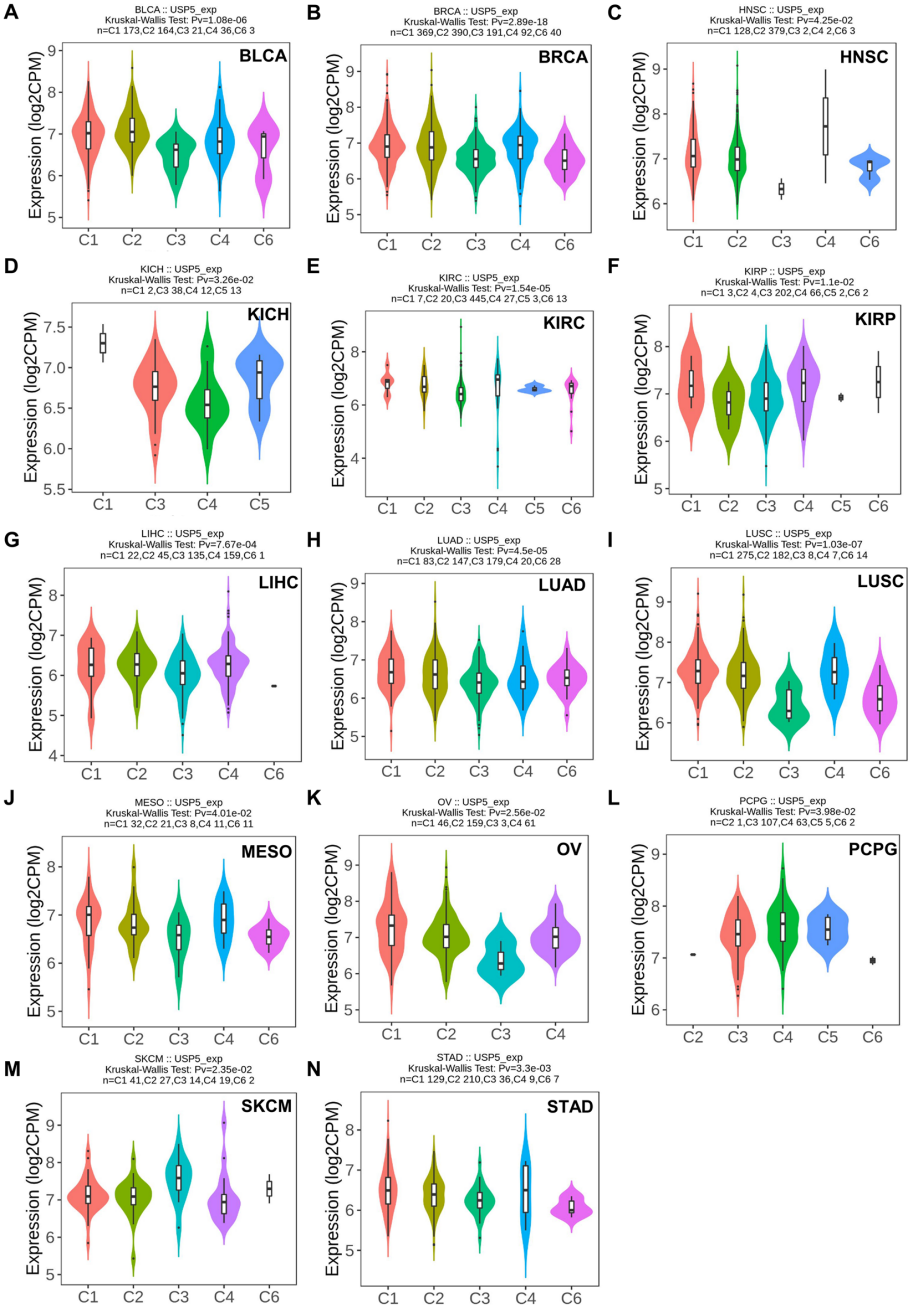
Correlations between USP5 expression and immune subtypes in pan-cancer. (**A**) BLCA. (**B**) BRCA. (**C**) HNSC. (**D**) KICH. (**E**) KIRC. (**F**) KIRP. (**G**) LIHC. (**H**) LUAD. (**I**) LUSC. (**J**) MESO. (**K**) OV. (**L**) PCPG. (**M**) SKCM. (**N**) STAD. Correlation analysis between USP5 expression and immune subtypes performed via TISIDB database.

We analyzed the correlation between USP5 expression and molecular or immune subtypes in pan-cancer from the TISIDB database. The results indicated that USP5 was expressed differently in 10 of 17 cancers for molecular subtypes, which showed the most increased level in the LumB subtype of BRCA (5 subtypes), HN-SNV subtype of COAD (4 subtypes), ESCC subtype of ESCA (5 subtypes), Classical subtype of HNSC (4 subtypes), C2b subtype of KIRP (4 subtypes), G-CIMP-low subtype of LGG (6 subtypes), primitive subtype of LUSC (4 subtypes), proliferative subtype of OV (4 subtypes), Kinasesignaling subtype of PCPG (4 subtypes) and CN_HIGH subtype of UCEC (4 subtypes) (Fig. 4A–J). Meanwhile, for immune subtypes (C1: wound healing, C2: IFN-gamma dominant, C3: inflammatory, C4: lymphocyte depleted, C5: immunologically quiet, C6: TGF-b dominant), we found that USP5 expression was significantly different in 14 of 30 cancers, including BLCA, BRCA, HNSC, KICH, KIRC, KIRP, LIHC, LUAD, LUSC, MESO, OV, PCPG, SKCM and STAD (Fig. 5A–N).

### Diagnostic value of USP5 in pan-cancer

**Figure 6:**
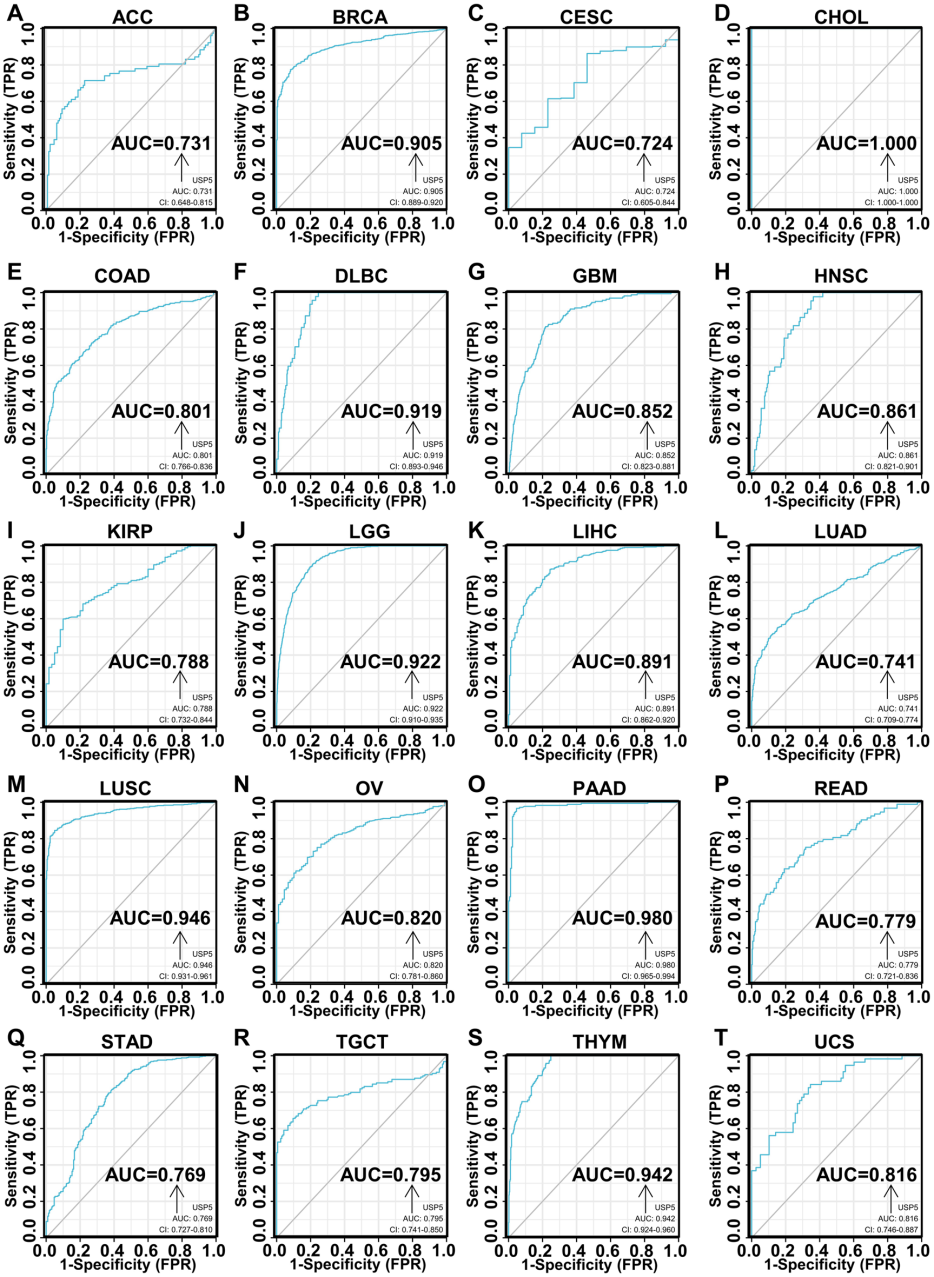
Receiver operator characteristic (ROC) analysis of USP5 in pan-cancer. (**A**) ACC. (**B**) BRCA. (**C**) CESC. (**D**) CHOL. (**E**) COAD. (**F**) DLBC. (**G**) GBM. (**H**) HNSC. (**I**) KIRP. (**J**) LGG. (**K**) LIHC. (**L**) LUAD. (**M**) LUSC. (**N**) OV. (**O**) PAAD. (**P**) READ. (**Q**) STAD. (**R**) TGCT. (**S**) THYM. (**T**) UCS.

We plotted the receiver operating curve (ROC) to investigate the diagnostic value of USP5 in pan-cancer. And the receiver operating curve analysis demonstrated that USP5 had certain diagnostic accuracy (the area under the curve > 0.7) in 20 cancer types, including ACC, BRCA, CESC, CHOL, COAD, DLBC, GBM, HNSC, KIRP, LGG, LIHC, LUAD, LUSC, OV, PAAD, READ, STAD, TGCT, THYM and UCS (Fig. 6A–T). Among them, USP5 had great diagnostic performance (the area under the curve > 0.9) in BRCA, CHOL, DLBC, LGG, LUSC, PAAD and THYM.

### Prognostic value of USP5 in pan-cancer

**Figure 7:**
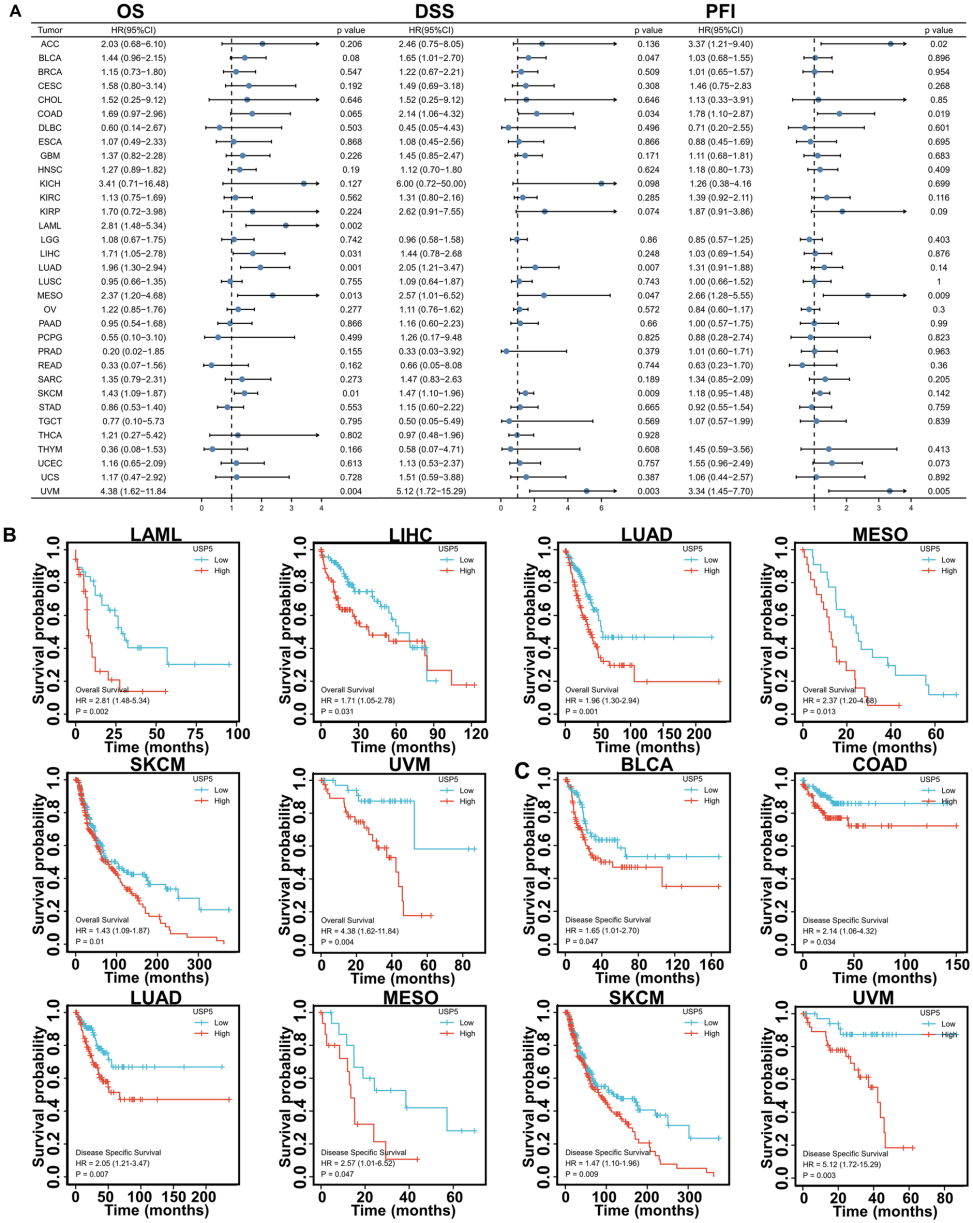

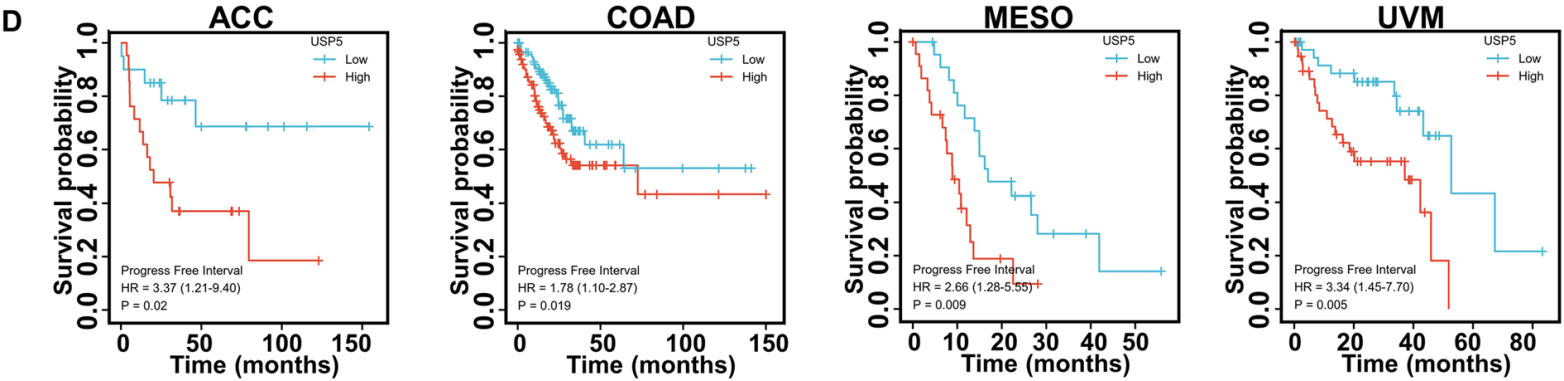
Correlation between USP5 expression and patient’s prognosis in pan-cancer. (**A**) Forest plots showed the correlation between USP5 expression and OS, DSS or PFI in different cancers. (**B**) Kaplan–Meier curves for patient’s overall survival classified by different expression level of USP5 in LAML, LIHC, LUAD, MESO, SKCM and UVM. (**C**) Kaplan–Meier curves for patient’s disease-specific survival classified by different expression level of USP5 in BLCA, COAD, LUAD, MESO, SKCM and UVM. (**D**) Kaplan–Meier curves for patient’s progress-free interval classified by different expression level of USP5 in ACC, COAD, MESO and UVM.

To evaluate the prognostic assessment value of USP5 in pan-cancer, we carried out the Cox proportional hazards model and Kaplan–Meier analysis. And the result showed that the high level of USP5 predicted poor overall survival of LAML, LIHC, LUAD, MESO, SKCM and UVM (Fig. 7A,B). For disease-specific survival, USP5 played a risk role for BLCA, COAD, LUAD, MESO, SKCM and UVM (Fig. 7A,C). Furthermore, patients with high expression of USP5 had shortened progress-free interval in ACC, COAD, MESO and UVM (Fig. 7A,D).

### Genetic alteration of USP5 in pan-cancer

**Figure 8:**
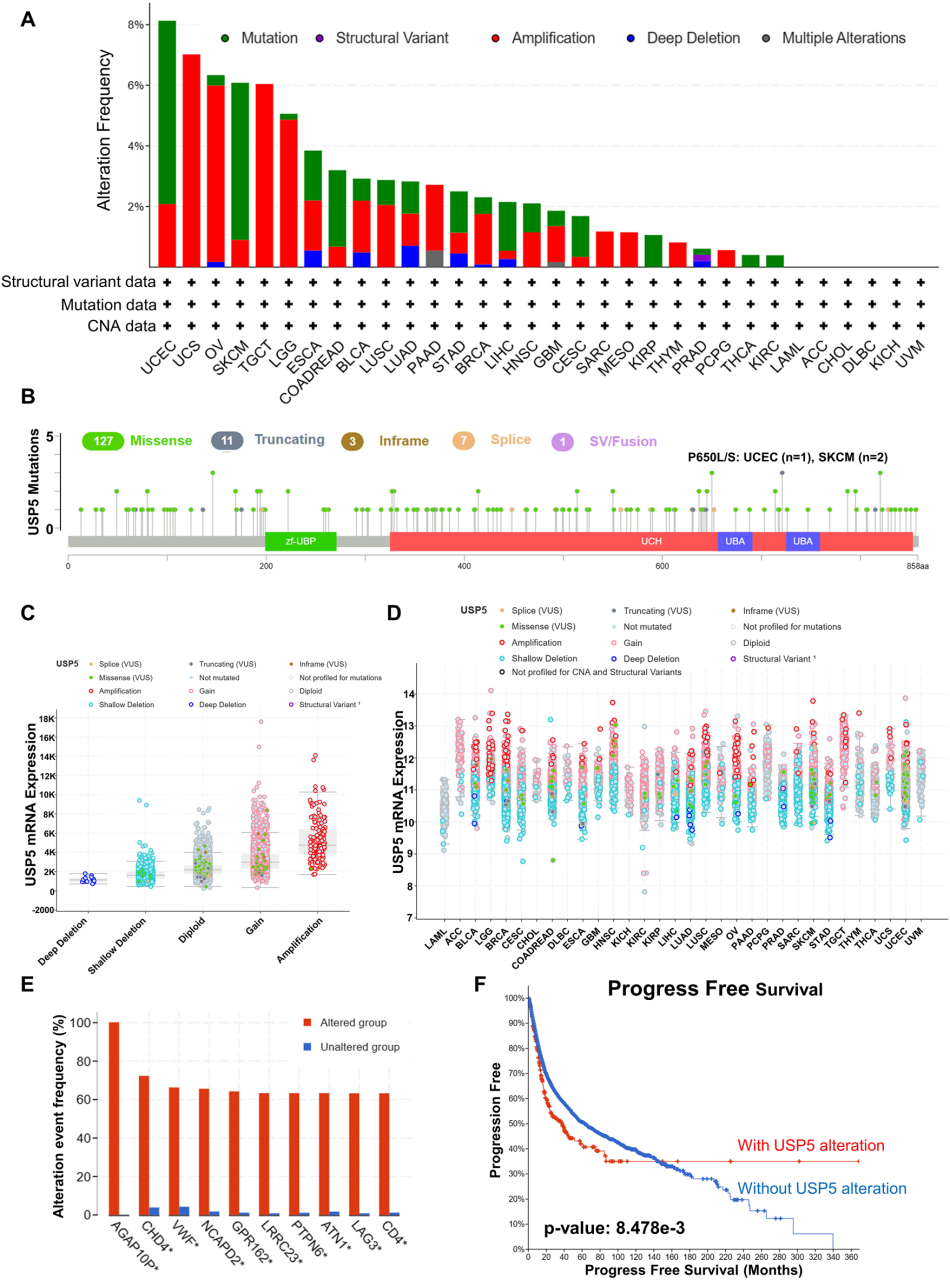
The genetic alterations of USP5 in pan-cancer. (**A**) Summary of USP5 genetic alteration in TCGA PanCancer Atlas Studies. (**B**) Mutation types, number and sites of USP5 across protein domains. (**C**,**D**) Correlation between the putative CNA of USP5 and its expression in cancers. (**E**) The related genes alteration frequency in groups with or without USP5 alteration. (**F**) Correlation between USP5 mutation status and PFS of cancer patients. The genetic mutations analysis of USP5 in pan-cancer was conducted by cBioPortal online platform based on TCGA data.

To investigate the genetic mutations of USP5 in pan-cancer, we used cBioPortal online platform based on TCGA data. The highest frequency of USP5 alteration appeared in UCEC, UCS, OV, SKCM, TGCT and LGG. And mutation, amplification and deep deletion were the most common genetic alterations types of USP5 (Fig. 8A). In addition, we found 149 mutation sites with missense mutation as the main alteration type in USP5. For instance, a missense mutation within the ubiquitin carboxyl-terminal hydrolase (UCH) damain, P650L/S alteration was detected in one case of UCEC and two cases of SKCM (Fig. 8B). Then the correlation between the putative CNA of USP5 and its gene expression in pan-cancer was shown in Fig. 8C,D. Moreover, compared with the unaltered group, the gene alteration of AGAP10P, CHD4, VWF, NCAPD2, GPR162, LRRC23, PTPN6, ATN1, LAG3 and CD4 was more predominant in group with USP5 alteration (Fig. 8E). Last, we studied the effect of USP5 genetic alteration on the prognosis of patients in pan-cancer, and the result indicated that patients with USP5 alteration had poor progress-free survival in pan-cancer (Fig. 8F), but not overall survival, disease-free survival and disease-specific survival (Supplementary Fig. 3A–C).

### Analysis of the methylation level of USP5 in pan-cancer

**Figure 9:**
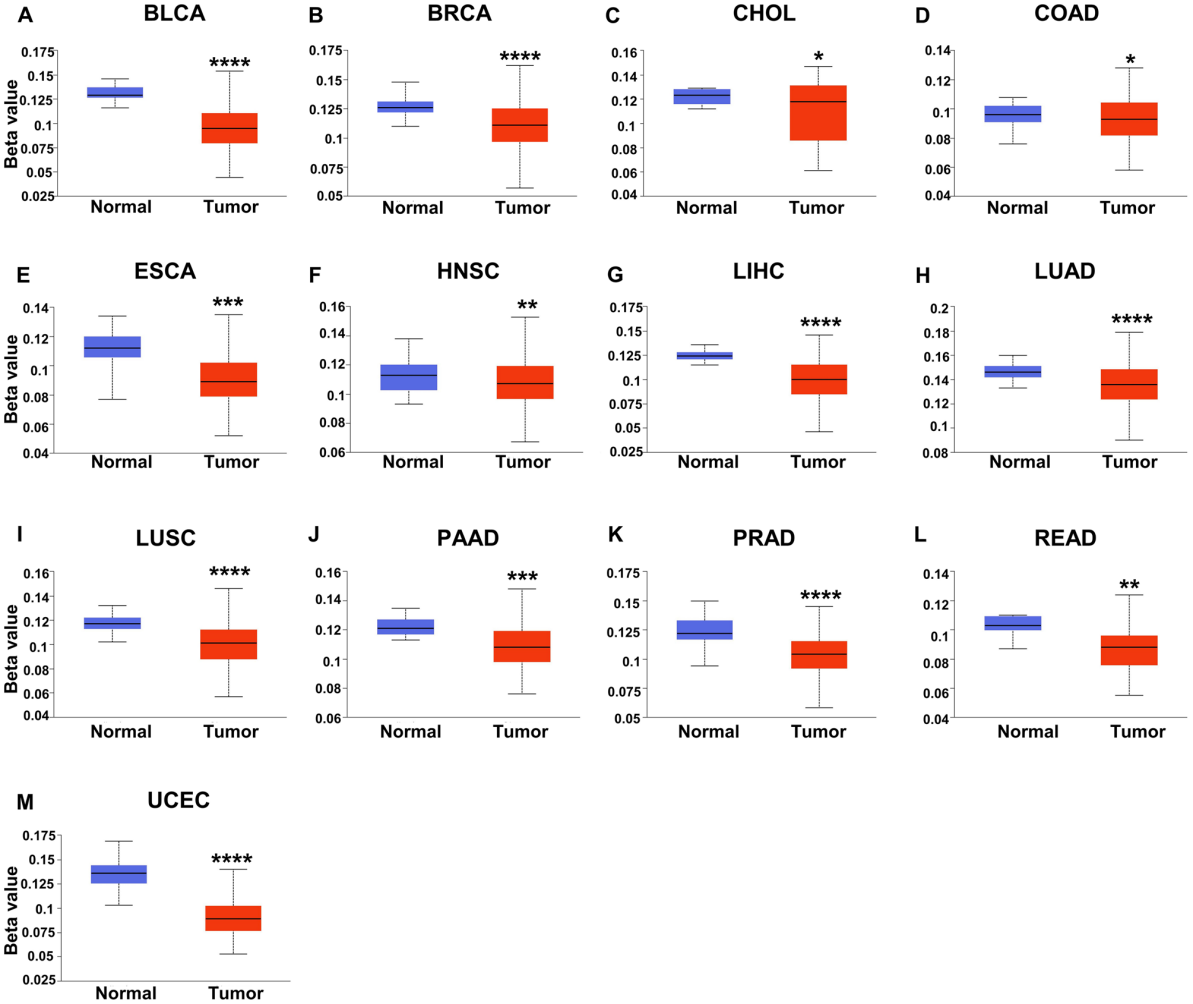
DNA methylation features of USP5 in pan-cancer. (**A**) BLCA. (**B**) BRCA. (**C**) CHOL. (**D**) COAD. (**E**) ESCA. (**F**) HNSC. (**G**) LIHC. (**H**) LUAD. (**I**) LUSC. (**J**) PAAD. (**K**) PRAD. (**L**) READ. (**M**) UCEC. The methylation level of USP5 obtained using UALCAN database.

DNA methylation has been proved to play an essential role in the occurrence and progression of cancers. Using UALCAN database, we compared the methylation level of USP5 between normal and cancer tissues. We found that the promoter methylation level of USP5 decreased in most cancers, including BLCA, BRCA, CHOL, COAD, ESCA, HNSC, LIHC, LUAD, LUSC, PAAD, PRAD, READ and UCEC (Fig. 9A–M). An obvious increase in the methylation level of USP5 was showed in KIRP, KIRC, TGCT and THCA (Supplementary Fig. 4A). And the difference of the USP5 methylation level was not significant in CESC, GBM, PCPG, SARC, STAD and THYM (Supplementary Fig. 4B).

### Immunogenomic analyses of USP5 in pan-cancer

**Figure 10:**
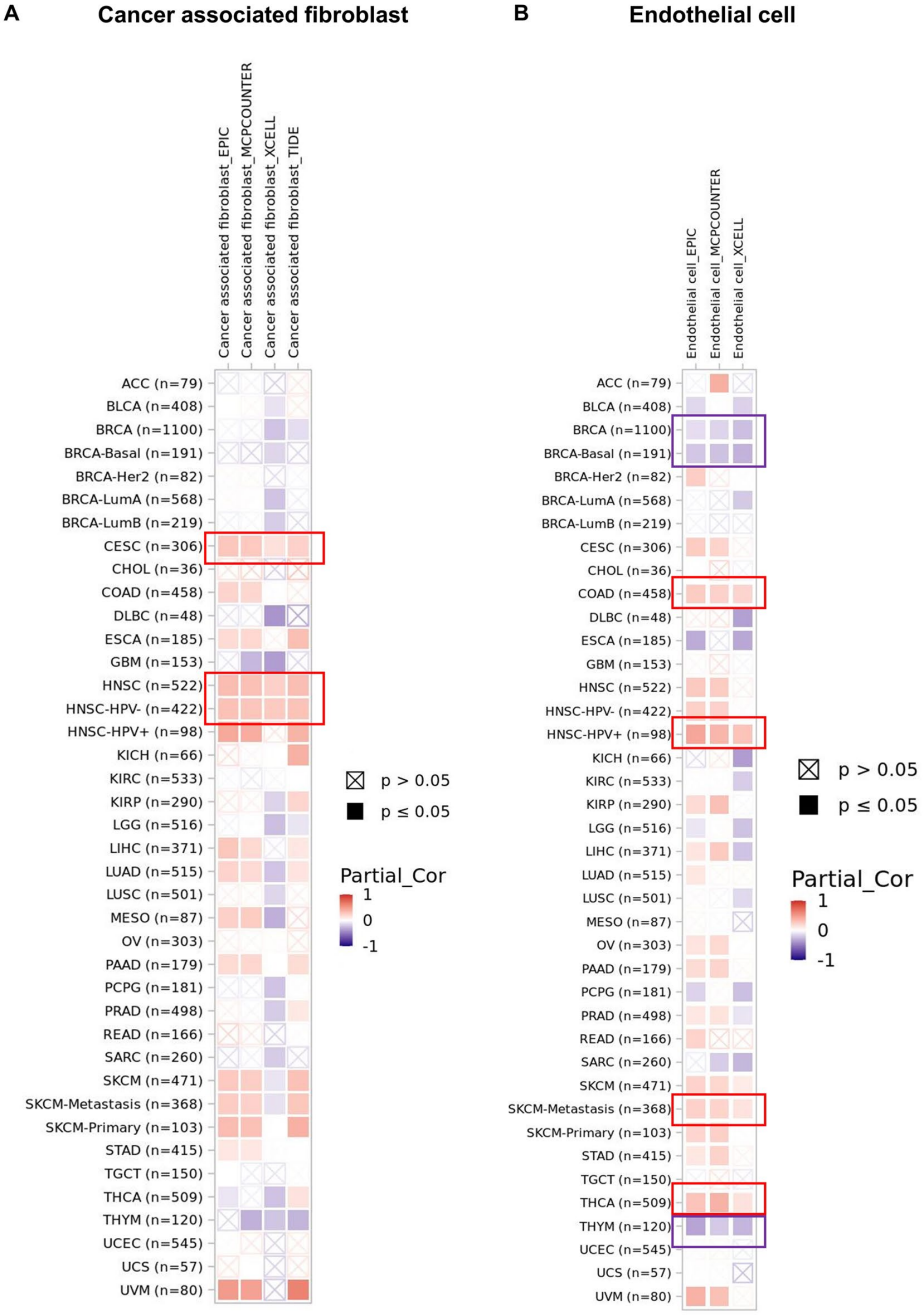
Correlation between USP5 expression and immune infiltrates in pan-cancer. (**A**,**B**) The relationship between USP5 expression level and infiltration of cancer-associated fibroblasts (**A**) and endothelial cells (**B**) across all TCGA tumors. The red square represented positive correlation (0–1), while blue square indicated negative correlation (− 1 –0). p value < 0.05 was considered as statistically significant. A cross mean non-significant correlations. The relationship between USP5 expression and immune infiltration levels across all TCGA cancers analyzed via TIMER2.0 tool.

Considering the critical role of immune infiltration and immune regulation in the oncology progress, we first applied CIBERSORT, CIBERSORT-ABS, EPIC, MCPCOUNTER, QUANTISEQ, TIDE, TIMER and XCELL algorithms to explore the correlation between USP5 expression and the infiltration level of different immune and endothelial cells in pan-cancer of TCGA. The result showed that USP5 expression was positively correlated with the infiltration of cancer-associated fibroblasts in CESC, HNSC and HNSC-HPV (Fig. 10A). In addition, we discovered a positive correlation between USP5 expression and endothelial cell infiltration in COAD, HNSC-HPV+, SKCM-Metastasis and THCA, while negatively correlated with endothelial cell infiltration in BRCA, BrCA-basal and THYM (Fig. 10B).

Also, we observed that USP5 was correlated with most immune inhibitors and immune stimulators except for KIR2DL1, KIR2DL3 and TNFSF18 in pan-cancer (Supplementary Fig. 5A,B). In terms of major histocompatibility complexes (MHCs), USP5 was positively associated with most major histocompatibility complexes in KIRC, KIRP and UVM, and negatively associated with most major histocompatibility complexes in ESCA, KICH, LUSC and TGCT (Supplementary Fig. 5C). Moreover, we found that USP5 showed certain correlation with majority of chemokines with the exception of CCL1, CCL16, CCL27, CCL24 and CCL25 in pan-cancer (Supplementary Fig. 5D). Meanwhile, a negative correlation between USP5 and most chemokine receptors could be found in the majority of malignant tumors especially in ESCA, KICH, LUSC and TGCT (Supplementary Fig. 5E).

### Functional states analysis of USP5 at single cell levels

**Figure 11:**
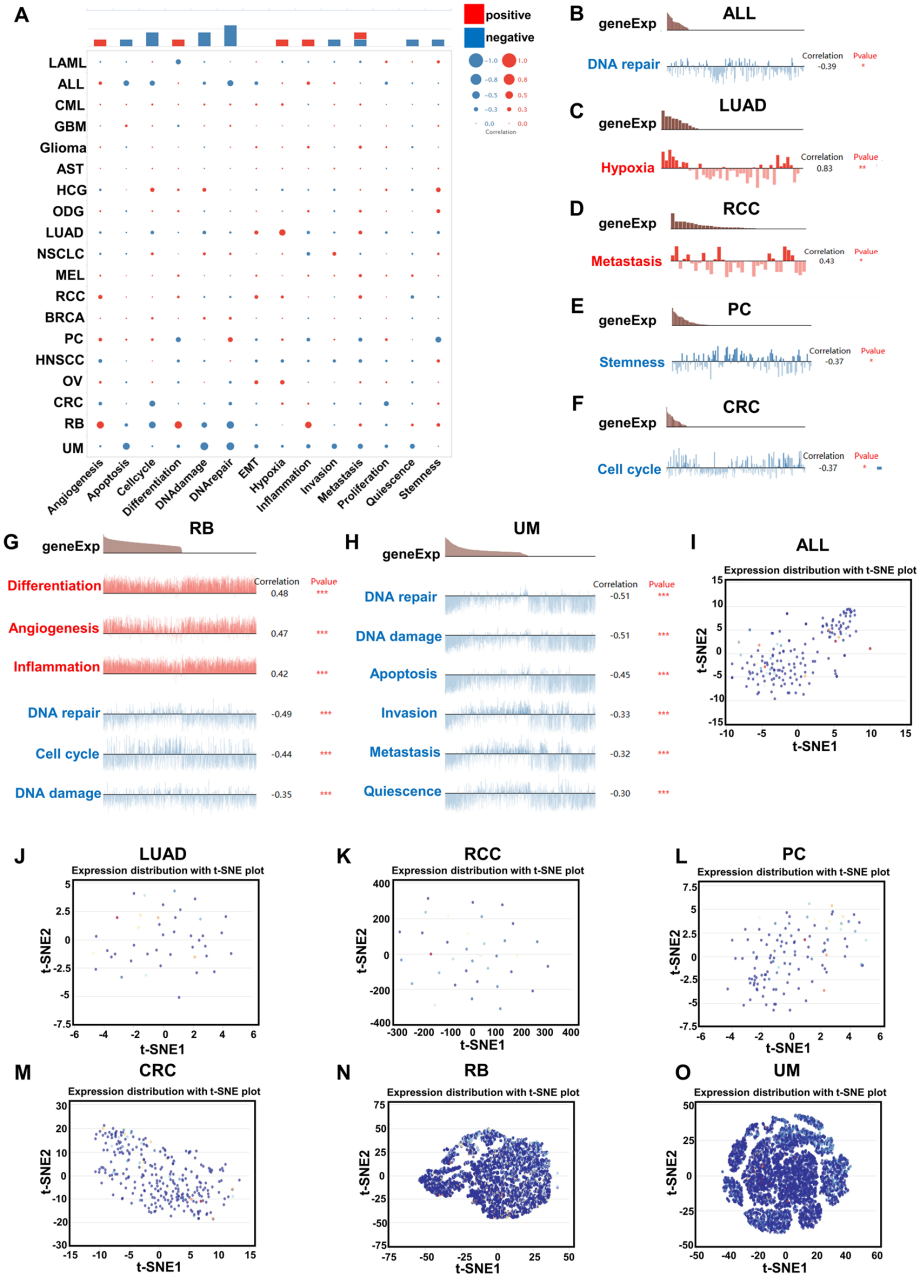
Correlation between USP5 expression and different functional states in pan-cancer. (**A**–**H**) The relationship of USP5 expression with functional states in cancers was explored via the CancerSEA website. (**I**–**O**) T-SNE diagram showed USP5 expression profiles in ALL (**I**), LUAD (**J**), RCC (**K**), PC (**L**), CRC (**M**), RB (**N**) and UM (**O**) at single cell levels. *p < 0.05, **p < 0.01, ***p < 0.001.

Using the CancerSEA, we investigated the functional states of USP5 at single cell levels in various cancers. The results indicated that USP5 was positively correlated with angiogenesis, differentiation, hypoxia, inflammation and metastasis, and negatively correlated with apoptosis, cell cycle, DNA damage, DNA repair, invasion, metastasis, quiescence and stemness (Fig. 11A). Then, the association between USP5 and specific cancer types was further examined. And we observed that USP5 showed positive correlation with hypoxia in LUAD; with metastasis in RCC; with differentiation, angiogenesis and inflammation in RB (Retinoblestoma). In contrast, USP5 negative correlated with DNA repair in ALL (Acute Lymphoblastic Leukemia); with stemness in PC (Prostate cancer); with cell cycle in CRC (Colorectal cancer); with DNA repair, cell cycle and DNA damage in RB; with DNA repair, DNA damage, apoptosis, invasion, metastasis and quiescence in UM (Uveal Melanoma) (Fig. 11B–H). Additionally, T-SNE diagrams were used to display USP5 expression profiles at single cell levels from ALL, LUAD, RCC, PC, CRC, RB and UM (Fig. 11I–O).

### Functional enrichment analysis of USP5 in pan-cancer

**Figure 12:**
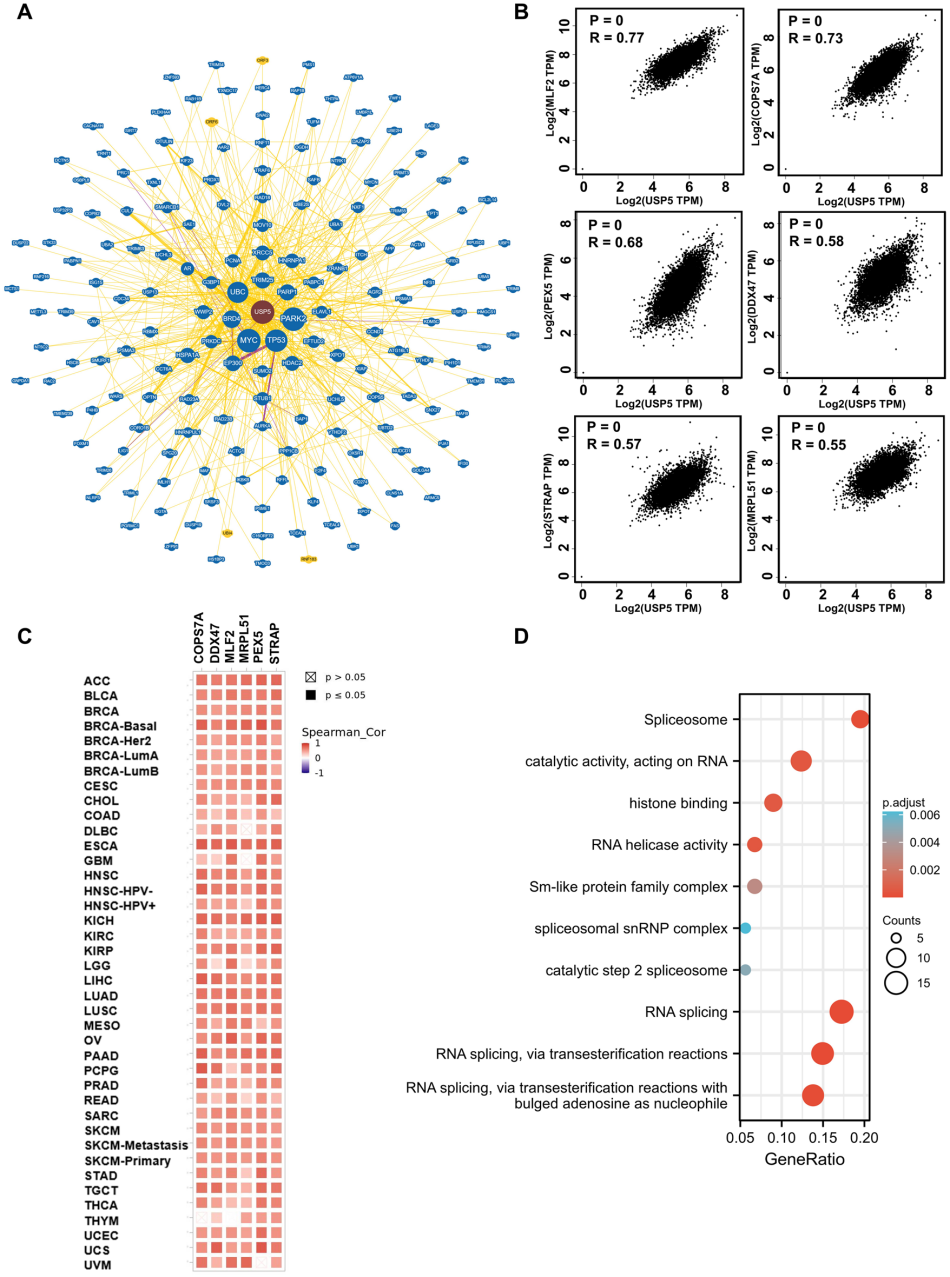
USP5-related genes functional enrichment analysis. (**A**) BioGRID web platform was used to get USP5-interacted molecules. (**B**) GEPIA2.0 showed the correlation between USP5 and six representative genes (MLF2, COPS7A, PEX5, DDX47, STRAP and MRPL51) of the USP5-related genes. p value < 0.001. (**C**) The heatmap confirmed the positive correlation between USP5 and MLF2, COPS7A, PEX5, DDX47, STRAP and MRPL51 in pan-cancer. (**D**) GO and KEGG enrichment analyses of USP5-related genes.

Finally, we screened out the USP5 co-expressed genes for a series of pathway enrichment analyses to understand the molecular mechanism of the USP5 gene in carcinogenesis and development. We first collected 179 molecules that interacted with USP5 via the BioGRID web service (Fig. 12A). Then we used GEPIA to acquire the top 100 USP5 co-expressed genes in pan-cancer. Among these, MLF2, COPS7A, PEX5, DDX47, STRAP and MRPL51 displayed strong correlations with USP5 in most cancer types (Fig. 12B,C). Furthermore, GO and KEGG enrichment analyses were used to reveal that USP5 co-expressed genes play a critical role in the regulation of spliceosome, RNA splicing, catalytic activity acting on RNA and histone binding in tumor pathogenesis (Fig. 12D).

## Discussion

Emerging studies indicated that USP5, a unique member of DUBs that can specifically recognize unanchored polyubiquitin, play an essential role in regulating the repair of DNA double-strand breaks^10^, inflammatory responses^11^, and stress responses^12^. Meanwhile, USP5 could regulate the stability of many tumorigenesis-associated proteins to influence the progression of various cancers such as, hepatocellular carcinoma^17^, pancreatic ductal adenocarcinoma^20,21^, and non-small cell lung cancer (NSCLC)^14,15^. However, the significance of USP5 in pan-cancer has not been explored until now. In the present study, using multiple bioinformatics approach, we first revealed the abnormal expression of USP5 in human cancers and its expression level in different molecular and immune subtypes of cancers, then we explored the diagnostic and prognostic values of USP5 in various cancers. In addition, we analyzed the gene mutations and methylation levels of USP5 in pan-cancer. Furthermore, the correlation between USP5 expression and the infiltration levels of immune cells and regulators was investigated, and the underlying functions of USP5 at single-cell levels was also identified. Finally, we implemented the functional enrichment analysis to recognize the potential mechanisms for USP5 to influence the pathogenesis of cancers.

Many studies revealed that USP5 was overexpressed and closely correlated with occurrence and progression of various cancers^18^. In line with their researches, our findings from TCGA and GTEx also demonstrated that the expression level of USP5 was significantly higher in most cancers compared with their regular counterparts. In addition, we found that there were meaningful correlations between USP5 expression level and the different molecular or immune subtypes of cancers, which suggested to us to get a deeper understanding of USP5’s function in cancer by targeting specific molecular or immune subtypes.

Based on our results, USP5 had a certain diagnostic accuracy (AUC > 0.7) in 20 cancer types, especially in predicting BRCA, CHOL, DLBC, LGG, LUSC, PAAD and THYM (AUC > 0.9), indicating the potential clinical application value of USP5 as a reliable diagnostic biomarker. Meanwhile, as a unique member of deubiquitinating enzymes (DUBs), USP5 could manage the stability of some key tumor-regulating proteins such as, Hypoxia-inducible factor 2α (HIF2α) in BRCA^13^, CyclinD1 (CCND1) in GBM^22^, Snail Family Transcriptional Repressor 2 (SNAI2) in LIHC^17^, Histone Deacetylase 2 (HDAC2) in OV^23^, Signal Transducer And Activator Of Transcription 3 (STAT3)^20^, WT1 Transcription Factor (WT1)^21^ and Forkhead Box M1 (FOXM1)^24^ in PAAD and Catenin Beta 1 (CTNNB1)^25^, Programmed cell death ligand 1 (PD-L1)^26^ and CyclinD1 (CCND1)^15^ in NSCLC to promote cancer progression, and our receiver operator characteristic (ROC) curve analysis showed the area under the curve (AUC) of 0.905 in BRCA, 0.852 in GBM, 0.891 in LIHC, 0.820 in OV, 0.980 in PAAD and 0.946 in LUSC. Thus, the combination of USP5 with these tumor-related biomarkers separately may significantly improve the diagnostic accuracy for the above cancers. Using cox proportional hazards model and Kaplan–Meier analysis, we found that USP5 was negatively correlated with patients’ prognosis generally. Specifically, USP5 expression indicated poor overall survival in patients with LAML, LIHC, LUAD, MESO, SKCM and UVM. In addition, we further analyzed the association between the expression level of USP5 and disease-specific survival or progress-free interval of cancer patients, and we proved that on the whole, USP5 exhibited risk role in MESO and UVM for overall survival, disease-specific survival and progress-free interval, in LUAD and SKCM for overall survival and disease-specific survival and in COAD for disease-specific survival and progress-free interval. In addition to the previous reported negative association between USP5 and the progression of BRCA^13^, BLCA^27^, CRC^16^, GBM^22^, LIHC^17^, melanoma^28^, NSCLC^15,26^, OV^23^ and PAAD^20^, our result first showed that USP5 may emerge as a novel biomarker for predicting the prognosis of ACC, LAML and MESO, especially MESO. These results suggested that USP5 had important diagnostic and prognostic implications in various cancers, and may serve as a therapeutic target for precision oncology.

USP5 gene was located in 12p13.31. The mutation of USP5 had been reported to cause several tissue disorders in drosophila, including severe defects in the eye development^29,30^. And the active site (C335A) mutation was proved to prevent the deubiquitination activity of USP5^31^. However, the studies on the USP5 gene alteration in human cancers were still rare. Here, we observed that USP5 genetic alterations, including mutation and amplification could be found in various cancer types. And the frequency of CHD4, VWF, NCAPD2, GPR162, LRRC23, PTPN6, ATN1, LAG3 and CD4 alterations was obviously higher in USP5 alteration group. Moreover, the patients with USP5 alteration had shorten progress-free survival in pan-cancer. DNA methylation, a common type of epigenetic modifications, generally inhibiting gene expression via altering chromatin structure, DNA stability, and DNA conformation, played an essential role in multiple types of tumorigenesis^32,33^. In this study, evidence indicated that DNA methylation level of USP5 was down-regulated in the majority of common malignancies, which was consistent with the elevation of USP5 expression. Further studies on the gene alteration of USP5 and the relationship between DNA methylation and USP5 expression in cancer are needed.

Tumor immune microenvironment (TIME), an essential part of tumor microenvironment (TME), mainly composed of immune cells, played a critical role in cancer progression^34–36^. Identifying new targets for immunotherapy was important for improving clinical outcomes, and the impact of USP5 on the TIME was rarely explored so far. The infiltrating immune cells were closely correlated with tumor growth, metastasis and invasion^37,38^. For example, cancer-associated fibroblasts, tumor-activated fibroblasts, could promote tumor development by secreting various cytokines or metabolites and forming barrier by shaping external-cellular matrix to inhibit the function of drugs and immune cells^39,40^. In addition, the proliferation of tumor endothelial cells had a protective function of tumor cells by preventing the blood lymphocytes from leaking out of the blood vessels and transporting them to the tumor^41^. And various immune cells recruited by progressed tumors can affect tumor growth, invasion and pathological angiogenesis by promoting the secretion of cytokines and chemokines^42,43^. In this study, we conducted analysis to assess the impact of USP5 on immune infiltration. And the result obtained through a variety of immune deconvolution methods showed that USP5 was significantly associated with the infiltration of immune cells, including CAFs and EC in certain tumors. Meanwhile, certain correlations between USP5 and various immunoregulation-related genes were found in many cancer types. Generally, our study suggested the potential value of USP5 as an effective target for immunotherapy to enhance the health of cancer patients. More preclinical and clinical trials are needed to explore the relationship between USP5 expression and immune checkpoints.

There was no doubt that USP5 played an essential role in tumorigenesis, but the underlying mechanism still remains elusive. Single-cell transcriptome sequencing was the key technique to analyze the potential functions of molecules at single-cell levels^44^. Using CancerSEA, we found that USP5 significantly correlated with many biological behaviors of cancers such as apoptosis, cell cycle, DNA damage, metastasis and invasion in several cancer types at single cell levels. Additionally, via the functional enrichment analyses of USP5 co-expressed genes, we showed that “spliceosome” and “RNA splicing” may be the critical mechanism for USP5 to involve in pan-cancer. Previous studies had demonstrated that USP5 could regulate cancers by mediating epithelial–mesenchymal transition, such as, in LIHC by stabilizing Snail Family Transcriptional Repressor 2^17^, in NSCLC by stabilizing Catenin Beta 1^25^ and in BRCA by stabilizing Hypoxia-inducible factor 2α ^13^. In addition, it had been found that silencing of USP5 may increase apoptosis and DNA damage to suppress the progression of PAAD^45^. Moreover, USP5 could promote cell cycle progression by preventing HERC4-mediated polyubiquitination of c-Maf in multiple myeloma^46^, and the downregulation of USP5 lead to the cell cycle arrest in UCEC^47^. Aberrant RNA splicing was thought to be critical in tumorigenesis^48^. In GBM, USP5 isoform2 was closely associated with the aberrant expression of polypyrimidine tract-binding protein 1 (PTBP1), an RNA splicing factor in GBM, and the forced expression of USP5 isoform1 inhibited cell growth and migration in two GBM cell lines, implying an essential role of individual USP5 isoforms generated by alternative splicing in gliomagenesis^49^. The potential molecular mechanism of USP5 related to tumorigenesis and whether USP5 could be a target for cancer therapy still need more experimental exploration.

In summary, using comprehensive bioinformatics analysis methods, our study explored the expression levels, potential diagnostic and prognostic value, genetic mutation, protein methylation, immunomodulatory effects and relevant signaling pathways of USP5 in pan-cancer. The results indicate that USP5 is overexpressed and has certain diagnostic value in various cancer types. In addition, USP5 may be a potential prognostic and immune-related biomarker for cancer patients. This study clarifies the role of USP5 in tumorigenesis from multiple perspectives, providing some bases for further research on the specific mechanisms of USP5 in the progression and treatment of cancers.

## Supplementary Information

Supplementary Information.

## Data Availability

The names of original contributions in this study are included in the article/supplementary material. Transcriptome data of 33 cancers and corresponding normal samples were obtained from TCGA and GTEx using the UCSC Xena platform (https://xenabrowser.net/datapages/). Other datasets generated and/or analysed during the current study are available in the following repository (http://gepia2.cancer-pku.cn/#analysis, https://www.proteinatlas.org/humanproteome/pathology, http://cis.hku.hk/TISIDB/, https://www.cbioportal.org/, http://ualcan.path.uab.edu/analysis.html, http://timer.cistrome.org/, http://biocc.hrbmu.edu.cn/CancerSEA/, https://thebiogrid.org/).
