# Supplementary material for: A pan-cancer analysis of the role of USP5 in human cancers
Source: Sci Rep. 2023 Jun 2;13:8972. doi: 10.1038/s41598-023-35793-2 (PMC10238416; doi:10.1038/s41598-023-35793-2)
Supplement: Supplementary file 1 — Supplementary Information. [file 41598_2023_35793_MOESM1_ESM.docx]

Supplementary Materials for

**A pan-cancer analysis of the role of USP5 in human cancers**

Bokang Yan^1^, Jiaxing Guo^2^, Shuang Deng^1^, Dongliang Chen^1^*, Meiyuan Huang^1^*

*Correspondence: Meiyuan Huang [(meiyuanhuang2022@163.com)](mailto:(1156080708@qq.com)) or Dongliang Chen (1156080708@qq.com)

**This file includes:**

Figures S1 to S5

Table S1


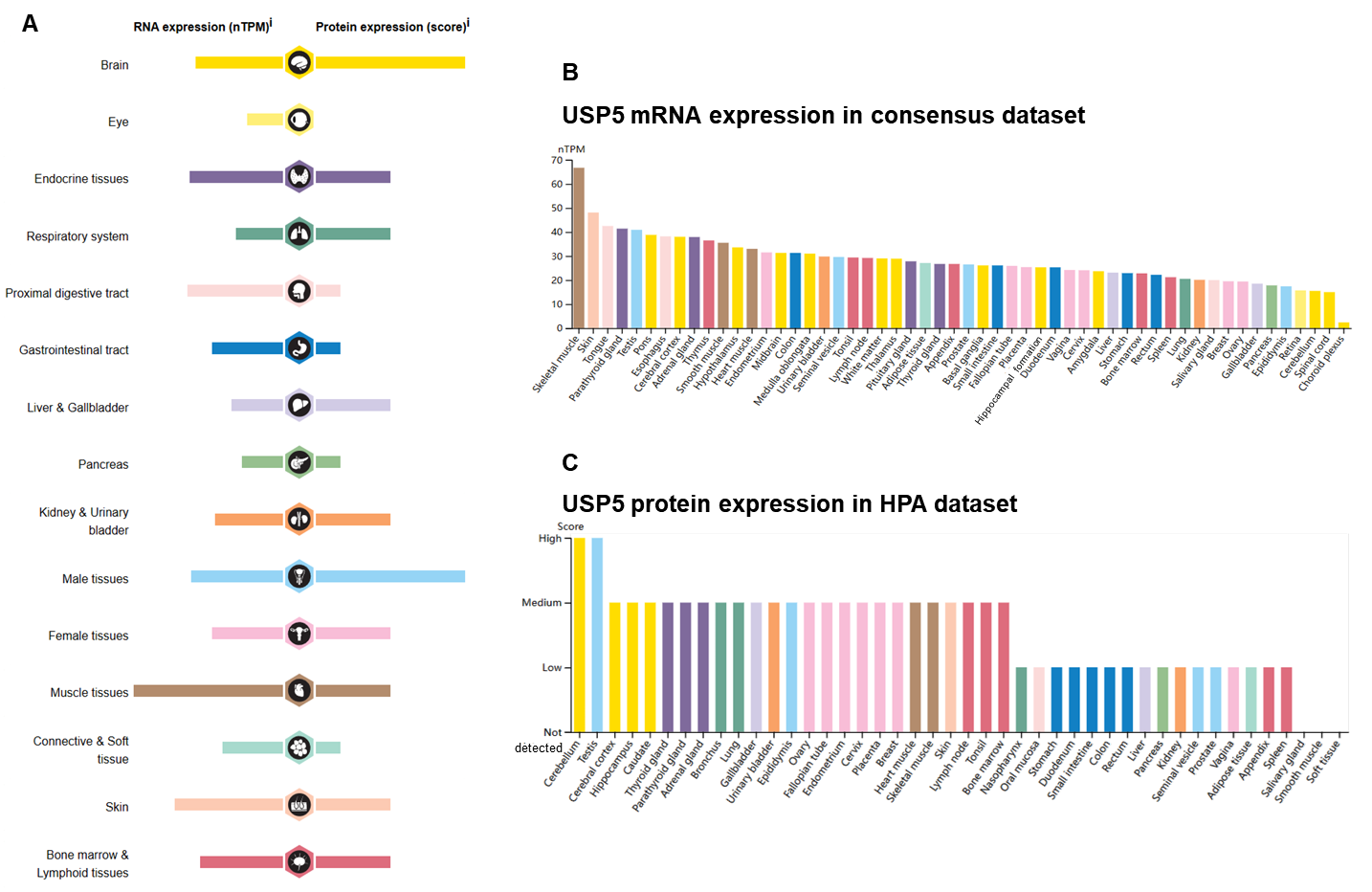


**Figure S1. The mRNA and protein expression levels of USP5 in human organs/tissues analyzed via HPA.** (A) The summary of USP5 mRNA and protein expression levels in human organs or tissues. (B) The expression summary of USP5 mRNA in various human organs or tissues. (C) The expression summary of USP5 protein in various human organs or tissues.

**
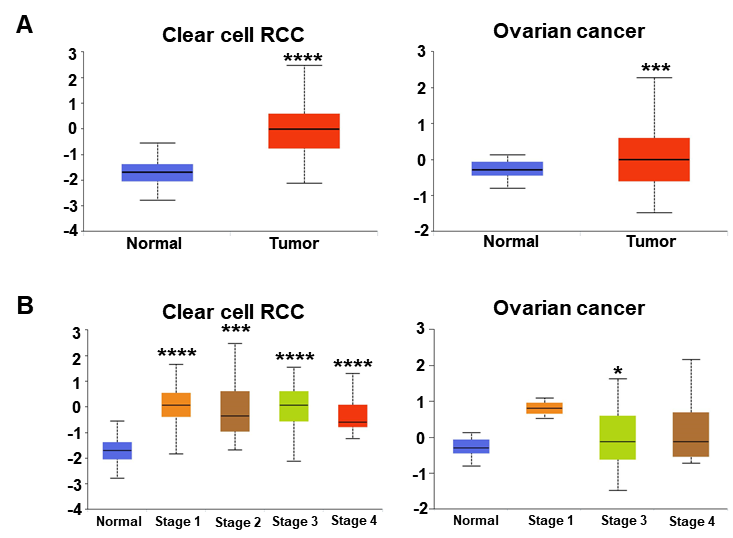
**

**Figure S2. The protein expression level of USP5 in different cancers.** (A) Using CPTAC web tool, the protein expression of USP5 was analyzed in clear cell RCC and ovarian cancer. (B) The protein expression of USP5 was analyzed by the main pathological stages of clear cell RCC and ovarian cancer.

**
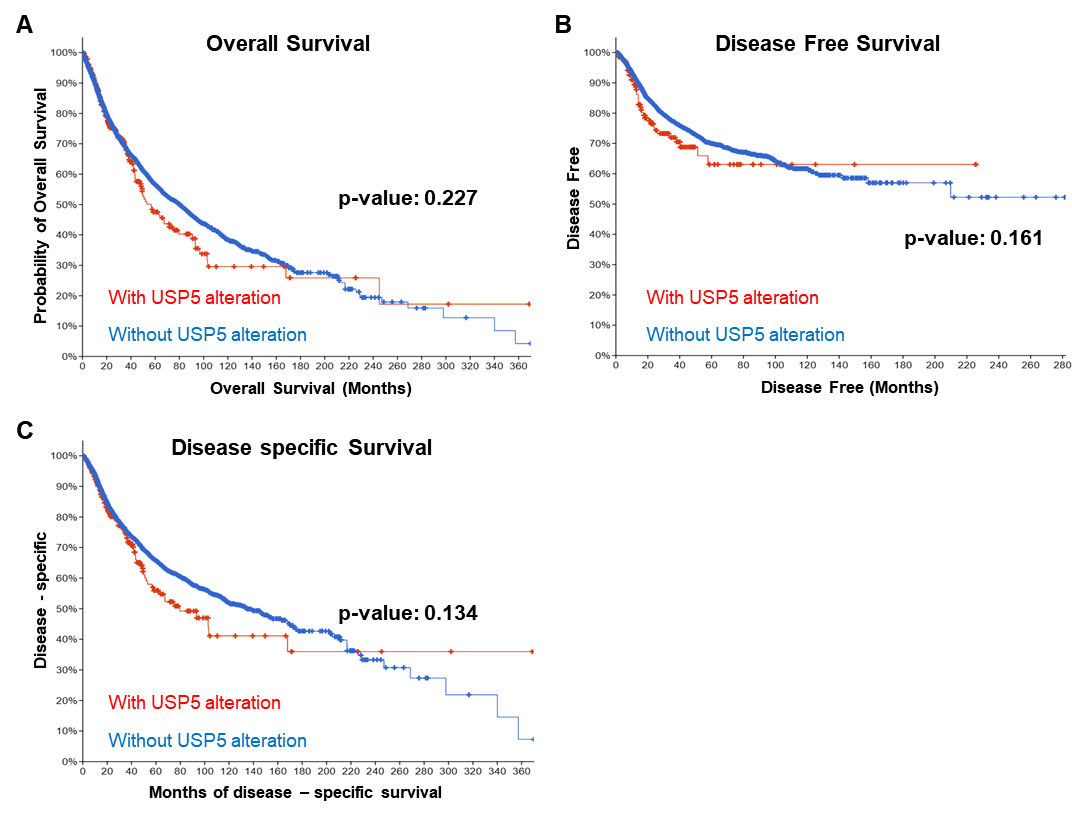
Figure S3. Analysis of the correlation between USP5 mutation status and patients’ prognosis of pan-cancer.** Correlations between USP5 mutation status and OS (A), DFS (B) and DSS (C) of cancer patients were analyzed on cBioPortal online platform based on TCGA data.

**
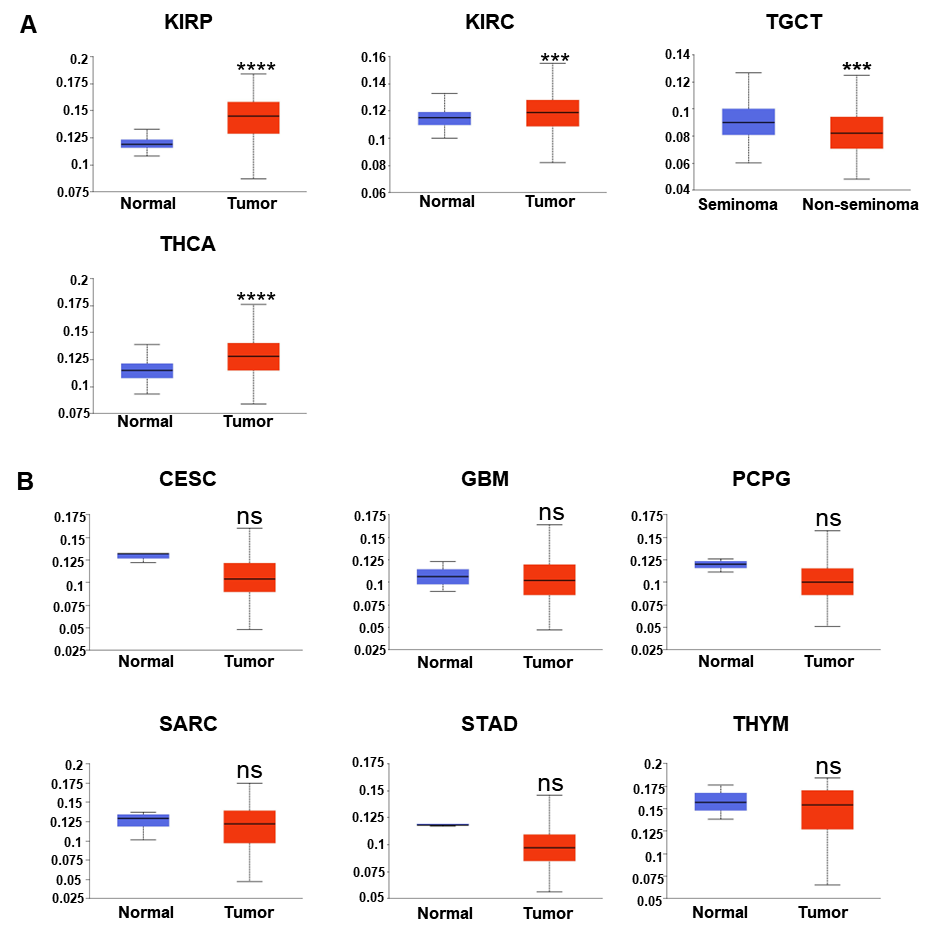
Figure S4. DNA methylation features of USP5 in pan-cancer.** (A) KIRP, KIRC, TGCT and THCA. (B) CESC, GBM, PCPG, SARC, STAD and THYM. The methylation level of USP5 obtained from UALCAN database.

**
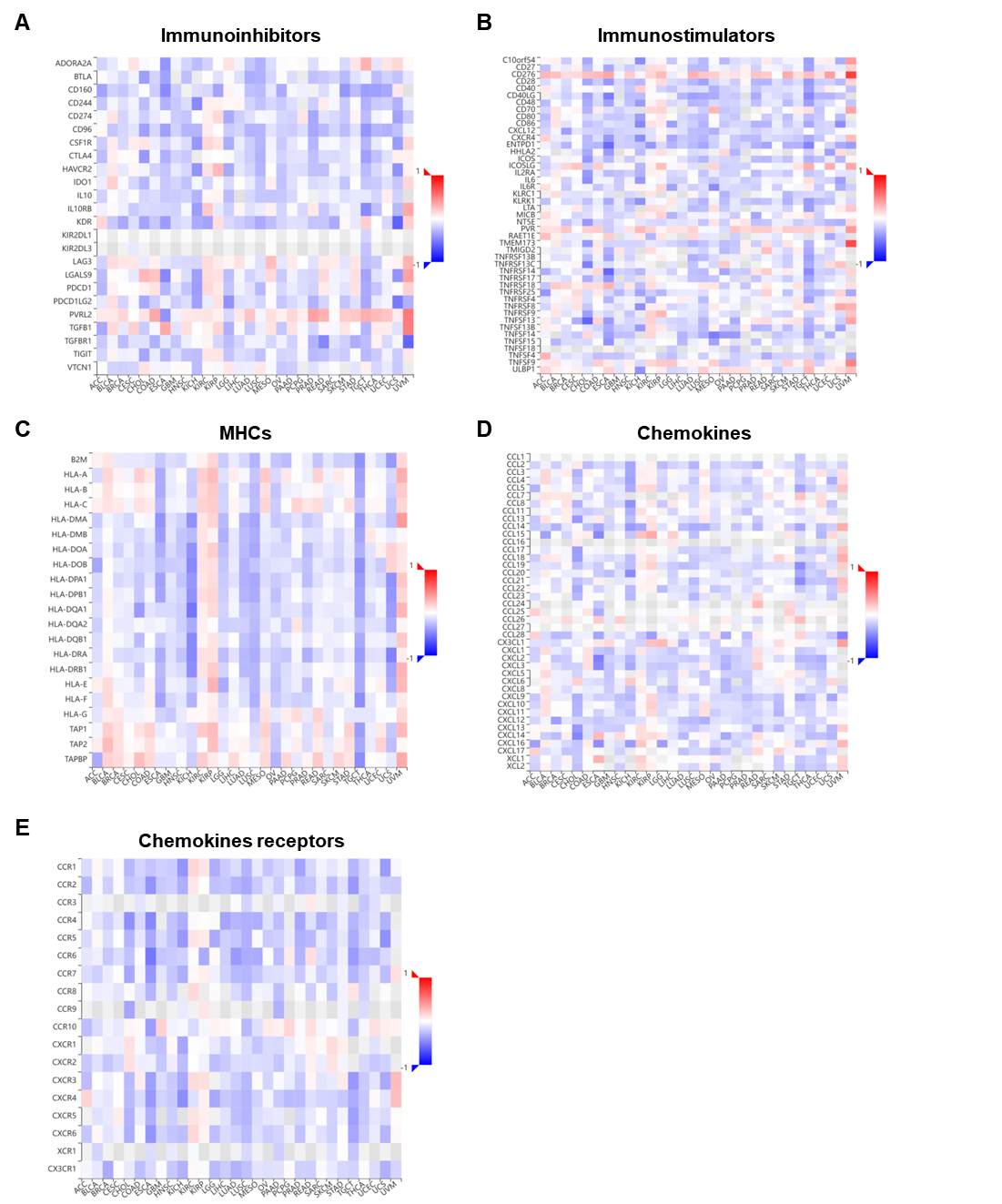
Figure S5. Correlation between USP5 and immunoregulation-related genes in pan-cancer.** Correlations between USP5 expression and immunostimulators (A), immunoinhibitors (B), MHC molecules (C), chemokines (D) and chemokine receptors (E) were obtained via the TISIDB database.

**Table S1. The abbreviation of 37 cancer types analyzed in this study.**

| **Abbreviation** | **Cancer types** |
| --- | --- |
| ACC | Adrenocortical carcinoma |
| ALL | Acute Lymphoblastic Leukemia |
| BLCA | Bladder Urothelial Carcinoma |
| BRCA | Breast invasive carcinoma |
| CESC | Cervical squamous cell carcinoma and endocervical adenocarcinoma |
| CHOL | Cholangiocarcinoma |
| COAD | Colon adenocarcinoma |
| COADREAD/CRC | Colorectal cancer |
| DLBC | Lymphoid Neoplasm Diffuse Large B-cell Lymphoma |
| ESCA | Esophageal carcinoma |
| GBM | Glioblastoma multiforme |
| HNSC | Head and Neck squamous cell carcinoma |
| KICH | Kidney Chromophobe |
| KIRC | Kidney renal clear cell carcinoma |
| KIRP | Kidney renal papillary cell carcinoma |
| LAML | Acute Myeloid Leukemia |
| LGG | Brain Lower Grade Glioma |
| LIHC | Liver hepatocellular carcinoma |
| LUAD | Lung adenocarcinoma |
| LUSC | Lung squamous cell carcinoma |
| MESO | Mesothelioma |
| OV | Ovarian serous cystadenocarcinoma |
| PAAD | Pancreatic adenocarcinoma |
| PCPG | Pheochromocytoma and Paraganglioma |
| PRAD/PC | Prostate adenocarcinoma |
| RB | Retinoblestoma |
| RCC | Renal cell carcinoma |
| READ | Rectum adenocarcinoma |
| SARC | Sarcoma |
| SKCM | Skin Cutaneous Melanoma |
| STAD | Stomach adenocarcinoma |
| TGCT | Testicular Germ Cell Tumors |
| THCA | Thyroid carcinoma |
| THYM | Thymoma |
| UCEC | Uterine Corpus Endometrial Carcinoma |
| UCS | Uterine Carcinosarcoma |
| UVM/UM | Uveal Melanoma |
